# Polyomavirus BK, BKV microRNA, and urinary neutrophil gelatinase-associated lipocalin can be used as potential biomarkers of lupus nephritis

**DOI:** 10.1371/journal.pone.0210633

**Published:** 2019-01-14

**Authors:** Yi-Jung Li, Hsin-Hsu Wu, Shou-Hsuan Liu, Kun-Hua Tu, Cheng-Chia Lee, Hsiang-Hao Hsu, Ming-Yang Chang, Kuang-Hui Yu, Wei Chen, Ya-Chung Tian

**Affiliations:** 1 Kidney Research Center and Department of Nephrology, Linkou Chang Gung Memorial Hospital, Taoyuan, Taiwan; 2 Department of Medicine, Chang Gung University, Taoyuan, Taiwan; 3 Graduate Institute of Clinical Medical Sciences, Chang Gung University, Taoyuan, Taiwan; 4 Department of Rheumatology, Allergy, and Immunology, Linkou Chang Gung Memorial Hospital, Taoyuan, Taiwan; 5 Department of Nephrology, Xiamen Chang Gung Hospital, Fujian Province, China; Istituto Di Ricerche Farmacologiche Mario Negri, ITALY

## Abstract

**Objective:**

Lupus nephritis (LN) frequently progresses to end-stage renal disease. Finding a biomarker for LN and a predictor for the development of chronic kidney disease (CKD) is important for patients with systemic lupus erythematosus (SLE).

**Methods:**

Ninety patients with SLE were divided into biopsy-proven LN (n = 54) and no kidney involvement (non-LN) (n = 36) groups and followed up for 54 months.

**Results:**

Of 36 patients with LN, 3 (5.6%) had class II disease, 3 (5.6%) had class III, 35 (64.8%) had class IV, 10 (18.5%) had class V, and 3 (5.6%) had class VI (advanced sclerosis). Compared to the non-LN group, patients in the LN group had higher autoimmunity evidenced by a higher proportion of low C3 and C4 levels, positive anti-double-stranded DNA antibody levels, and lower estimated glomerular filtration rates (eGFR). Urinary neutrophil gelatinase-associated lipocalin (uNGAL) levels were significantly higher in the LN group (LN vs non-LN, 670 vs 33 ng/mL, respectively). The patients with LN had a higher urinary polyomavirus BK (BKV) load (3.6 vs 3.0 log copies/mL) and a lower urinary BKV miRNA (miR-B1) 5p level (0.29 vs 0.55 log copies/mL, p = 0.025), while there was no significant difference in the level of miR-B1-3p. Urinary miR-B1-5p level but not urinary BKV load was negatively correlated with uNGAL level (r = -0.22, p = 0.004). At the cutoff value of 80 ng/mL, the receiver operating characteristic curve analysis showed that uNGAL level as a predictor of the presence of LN had a high sensitivity (98%) and specificity (100%) (area under the curve [AUC], 0.997; p < 0.001). During the 54-month follow-up period, 14 (7%) patients with LN and none of the non-LN patients developed CKD. Multivariate Cox regression analysis revealed that baseline uNGAL level was the only predictive factor for CKD development, while baseline serum creatinine level and eGFR were not.

**Conclusion:**

An elevated urinary BKV viral load with a decreased level of miR-B1 implies the presence of LN. In addition, an increased uNGAL level is a good biomarker not only in predicting the presence of LN but also for prediction of CKD development in patients with SLE.

## Introduction

Lupus nephritis (LN) is a serious complication of systemic lupus erythematosus (SLE) and often results in the progression to end-stage renal disease (ESRD) with high mortality. Despite advancement in new therapies, the treatment failure rate still remains high.[1] Unknown etiologies that affect SLE autoimmunity and difficulties with the early detection of renal flares may contribute to the high treatment failure rate.[2, 3] One possible solution is the discovery of biomarkers that can enable the early diagnosis of LN. Among the genetic and environmental factors that lead to LN development or deterioration, infections reportedly play a critical role in inducing flares.[4, 5] Epidemiological evidence has suggested that Epstein-Barr virus (EBV) and cytomegalovirus (CMV) might be risk factors for SLE.[6, 7] The concomitant treatment of CMV infection has been shown to cause remission of SLE activity.[6] Other latent viral infections such as herpes simplex virus and polyomavirus BK (BKV) are also among the culprits that trigger SLE autoimmunity.

BKV is an emerging human pathogen that has been identified to cause polyomavirus-associated nephropathy in kidney transplant recipients, which commonly leads to allograft failure.[8] Primary BKV infection is usually acquired in childhood and the virus then remains latent in the kidney.[9] BKV reactivation occurs frequently in immunocompromised patients, including transplant recipients, patients infected with human immunodeficiency virus (HIV), and those under immunosuppressive therapy. In bone marrow transplant recipients and HIV patients, BKV reactivation can cause hemorrhagic cystitis, pneumonitis, and encephalitis.[10, 11] Recent studies have also suggested that BKV may play a role in the pathogenesis of autoimmune diseases.[12]

BKV reactivation presenting as BK viruria and viremia have been reported in patients with SLE.[13] However, the association between BKV reactivation and LN is unclear. Some studies found that anti-double-stranded DNA antibody (anti-dsDNA) was frequently produced during BKV infection and that these autoantibodies could be considered a hallmark of LN.[14, 15] In addition, large T antigen (TAg) formations of BKV complexed with nucleosomes could be identified in renal specimens of patients with LN.[15] The findings of these human and animal studies suggest that BKV may be implicated in the pathogenesis of SLE and could be considered a biomarker of LN.

BKV-encoded microRNAs (miRNAs) target BKV TAg with complementarity to its mRNA.[16] We have previously demonstrated that BKV miRNA (miR-B1) suppressed TAg expression and resulted in the negative autoregulation of viral replication.[17] Urinary BKV miR-B1 level can be used as a biomarker of polyomavirus-associated nephropathy (PVAN) in kidney transplant recipients because its expression is upregulated in patients with PVAN.[17, 18] As BKV is possibly involved in the pathogenesis of SLE, it would be of interest to evaluate the expression of BKV miRNAs in patients with SLE.

Traditional risk factors for LN, including an elevated anti-dsDNA titer and decreased complement level are not prompt and sensitive markers for the early detection of active renal disease.[19] A new biomarker, urinary neutrophil gelatinase-associated lipocalin (uNGAL), has been reported to have a better correlation with renal flare. However, whether uNGAL can provide a predictive value for the prognosis of LN in patients with SLE remains undetermined.

In this study, we aimed to assess the value of urinary BKV load, BKV miRNAs and uNGAL in predicting the presence of LN and the development of chronic kidney disease (CKD).

## Materials and methods

### Patients and definitions

A total of 90 patients with SLE were recruited in this investigation from November 2008 to July 2013. All patients signed a written informed consent in this study. Ethics approval (No. 97-1739B) was obtained from the Medial Ethics Committee of Linkou Chang Gung Memorial Hospital. The diagnosis of SLE was based on the 1997 American College of Rheumatology revised classification criteria. These patients were classified as having biopsy-proven LN or no kidney involvement (non-LN). Fifty-four patients were diagnosed with LN based on histological findings according to the International Society of Nephrology and WHO criteria for LN. Thirty-six non-LN patients had no kidney involvement, defined as a normal serum creatinine level or eGFR > 90 mL/min and a normal urine sediment without casts, red blood cells, or pyuria and proteinuria < 0.5 g according to the SLE disease activity index. The patients’ demographic characteristics, clinical manifestation, and medications were recorded. Results of laboratory tests including full blood count, biochemical parameters, serum complement C3 and C4, and anti-dsDNA were collected for analysis. The eGFR was calculated using the Modification of Diet in Renal Disease Study equation. Urine samples were collected from all patients with SLE for the assessment of the expression of BK viruria and BKV miRNA, and the level of NGAL, when enrolled. During the 54-month follow-up period, renal function was regularly measured at the interval of 1–3 months. CKD was defined as an eGFR < 60 mL/min that persisted for > 3 months regardless of the presence or absence of histological kidney damage or proteinuria. ESRD was defined as an eGFR < 15 mL/min or the need for long-term dialysis.

### Quantitative measurement of urinary BKV load

Urinary BKV load was determined by a quantitative polymerase chain reaction (qPCR) as described previously.[20] Briefly, DNA was extracted from 200μL of urine sample, using a QIAamp DNA Mini Kit (Qiagen, Hilden, Germany) according to the manufacturer’s instructions. To determine viral load, BKV and cellular DNA were extracted from cell lysate using a QIAamp^®^ DNA Mini Kit (Qiagen, Hilden, Germany). qPCR was performed as conventional protocol. The BKV DNA was normalized by analyzing samples in parallel by the qPCR for the cellular glyceraldehyde-3-phosphate dehydrogenase DNA (a housekeeping gene). The limit of detection was 10 copies/mL of BKV DNA.

### Quantification of mature microRNAs-stem-loop PCR

The sample RNA was mixed with reverse transcriptase master mix from an RNA Reverse Transcription Kit (ABI). The specific loop reverse transcription (RT) primer was designed according to the miRNA database (5′-TCAACTGGTGTCGTGGAGTCGGCAATTCAGTTGAGGACTCTGG-3′; 5′-CTCAACTGGTGTCGTGGAGTCGGCAATTCAGTTGAGATGCTCTT-3′) and added to the mixture. Following the RT reaction, the circular DNA was mixed with Master Mix, forward primer, reverse primer, and RNase free water. The sequences of the PCR primers were miR-B1-3p 5′-CGGCGGTGCTTGATCCATGTCC-3′; miR-B1-5p 5′-CGGCGGATCTGAGACTTGGGAA-3′; and universal primer 5′-CTGGTGTCGTGGAGTCGGCAATTC-3′. Real-time PCR was performed according to the manufacturer’s instructions using an ABI-Prism 7700 with SYBR Green I as a double-stranded DNA-specific dye (PE-Applied Biosystems).

### Measurement of uNGAL

Ten milliliters of each urine sample was centrifuged at 3,000 rpm for 10 minutes to remove the cellular debris. The cleaned urine samples were stored at *−*80°C until measurement. The uNGAL concentration was quantified using commercial neutrophil gelatinase-associated lipocalin (NGAL) enzyme-linked immunosorbent assay kits (Quantikine; R&D Systems, Inc.) according to the manufacturer’s instructions.

### Statistical analysis

Continuous variables are expressed as mean ± standard deviations and categorical variables are expressed as number (%). All data were checked for normal distribution and equal variances before analysis. For comparisons among two patient groups, continuous variables were analyzed by Student’s *t*-test, whereas categorical variables were analyzed by the chi-square test or Fisher’s exact test. P values < 0.05 were considered statistically significant. Univariate Cox regression analysis was used to compare the frequencies of possible risk factors for CKD. To control for potential confounding factors, multivariate Cox regression analysis (enter method) was performed to analyze the factors identified as significant (P < 0.05) on univariate analysis that met the proportional hazards assumption. The accuracy of uNGAL and serum creatinine to discriminate between LN and non-LN was evaluated by receiver operating characteristic (ROC) curve. The correlation analysis used Spearman’s correlation coefficient. Values of p < 0.05 were considered statistically significant.

## Results

### Patient characteristics

A total of 90 patients with SLE were divided into two groups according to the presence or absence of LN. Fifty-four patients with biopsy-proven LN were classified into the LN group. Of these patients, 3 (5.6%) had class II LN, 3 (5.6%) had class III, 35 (64.8%) had class IV, 10 (18.5%) had class V, and 3 (5.6%) had class VI (advanced sclerosis). Thirty-six patients had a normal serum creatinine level and urine sediment without casts, red blood cells or pyuria and proteinuria < 0.5 g/day, which qualified as no renal involvement, and were classified into the non-LN group. Demographics and laboratory measurements are summarized in Table 1. There was a female predominance in both groups, but sex ratio of the groups was not significantly different. Patients in the LN group were younger and had higher autoimmune activity that presented as a higher proportion of low serum C3 and C4 levels and positive anti-dsDNA, as compared to non-LN patients. The mean albumin and hemoglobulin serum levels were lower in the LN group. There were no significant differences in white blood cell or platelet counts. Analysis of immunosuppressant use showed that more patients in the LN group took higher doses of steroids and hydroxychloroquine and a higher proportion of LN patients used potent immunosuppressants than non-LN patients. As expected, there was a significantly lower baseline serum creatinine level and eGFR and a higher frequency of proteinuria in the LN group.

**Table 1:** Patient characteristics by group.

	Total (N = 90)	LN (n = 54)	Non-LN (n = 36)	P value
**Age**	37.1 ± 1.3	32.8 ± 1.3	43.5 ± 2.1	<0.001
Female (%)	75 (83%)	48 (89%)	27 (75%)	0.15
WBC count (1,000/mL)	7.4 ± 0.3	7.4 ± 0.5	7.5 ± 0.4	0.823
Hemoglobin (g/mL)	11.5 ± 0.3	10.8 ± 0.3	12.6 ± 0.5	0.001
Platelet count (1,000/mL)	224 ± 8	217 ± 11	234 ± 12	0.297
Creatinine (mg/dL)	1.4 ± 0.2	1.8 ± 0.3	0.7 ± 0.0	<0.001
eGFR (mL/min)	86 ± 5	74 ± 7	106 ± 6	<0.001
Albumin (g/dL)	3.1 ± 0.1	2.8 ± 0.1	4.3 ± 0.1	<0.001
Low C3 (%)	54 (60%)	42 (78%)	12 (33%)	0.005
Low C4 (%)	36 (40%)	33 (61%)	3 (8%)	0.005
Positive anti-dsDNA (%)	42 (47%)	32 (59%)	10 (28%)	0.007
Proteinuria (mg/dL)	228 ± 35	445 ± 42	5 ± 3	<0.001
Biopsy-proven lupus nephritis		54		
Class II (%)		3 (5.6%)		
Class III (%)		3 (5.6%)		
Class IV (%)		35 (64.8%)		
Class V (%)		10 (18.5%)		
Class VI (%)		3 (5.6%)		
Potent immunosuppressants^a^ (%)	18 (20%)	18 (33.3%)	0 (0)	<0.001
Prednisolone (mg/day)	31 ± 7	46 ± 12	9 ± 1	0.003
Hydroxychloroquine (mg/day)	180 ± 20	130 ± 24	256 ± 30	0.001
Urinary NGAL (ng/mL)	400 ± 115	666 ± 188	23 ± 3	0.001
Urinary BKV copies (log)/mL	3.3 ± 1.0	3.5 ± 0.1	3.0 ± 0.2	0.008
BKV miR-B1-5p copies (log)/mL	0.39 ± 0.06	0.29 ± 0.07	0.55 ± 0.09	0.025
BKV miR-B1-3p copies (log)/mL	0.92 ± 0.11	0.78 ± 0.13	1.12 ± 0.19	0.141

Anti-dsDNA, anti-double-stranded DNA antibody; BKV, urinary polyomavirus BK; eGFR, estimated glomerular filtration rate; LN, lupus nephritis; NGAL, neutrophil gelatinase-associated lipocalin; WBC, white blood cell

^a^Including mycophenate mofetil, azathioprine, and cyclophosphamide

### Urinary BKV load is higher in patients with LN than in patients without LN

BKV viruria is commonly detected in kidney transplant recipients and immunocompromised patients.[17, 21] As patients with SLE are immunosuppressed and thus at high risk of BKV reactivation, we investigated urinary BKV load in patients with SLE with and without LN. The quantitative PCR results revealed that the mean urinary BKV load in the LN group was 3.6 log copies/mL, significantly higher than that (3.0 log copies/mL) in the non-LN group (Table 1). As BKV replication can be affected by the use of immunosuppressants, most of patients with LN had received the immunosuppressive therapy and might have a higher urinary BKV load. We therefore analyzed urinary BKV load in 25 SLE patients without the use of immunosuppressive therapy when urine samples were collected. The results still demonstrated a higher urinary BKV load in patients with LN when compared with patients without LN (the LN group versus non-LN group: 3.0 ± 0.1 versus 3.2 ± 0.2 log copies/mL, p = 0.029).

Since BK viruria is associated with renal impairment in transplant recipients,[21] we sought to determine the correlation between urinary BKV load and baseline eGFR or renal injury marker uNGAL. The results of Spearman’s correlation did not show a good correlation between urinary BKV load and eGFR (r = -0.12, p = 0.28). Similarly, there was no good correlation between urinary BKV load and the kidney injury marker uNGAL (r = -0.06, p = 0.56).

### Urinary BKV miR-B1 levels are lower in patients with LN and negatively correlate with uNGAL levels in patients with SLE

Several studies reported that polyomavirus-encoded microRNA was expressed in the urine of patients with BKV infection.[17, 18, 22] To investigate whether BKV miR-B1 expression was detected in patients with SLE with or without LN, we measured urinary BKV miR-B1 levels in the LN and non-LN groups. The mean expression levels of urinary miR-B1-5p and -3p in all patients with SLE were 0.39 log copies/mL and 0.92 log copies/mL, respectively. The mean expression level of miR-B1-5p was lower in the LN group than in the non-LN group (0.29 versus 0.55 log copies/mL, p = 0.025), while there was no significant difference in the level of miR-B1-3p (0.78 versus 1.12 log copies/mL, p = 0.141). In addition, miR-B1-5p and -3p levels were not significantly correlated with baseline eGFR values in either group (data not shown).

**Fig 1:**
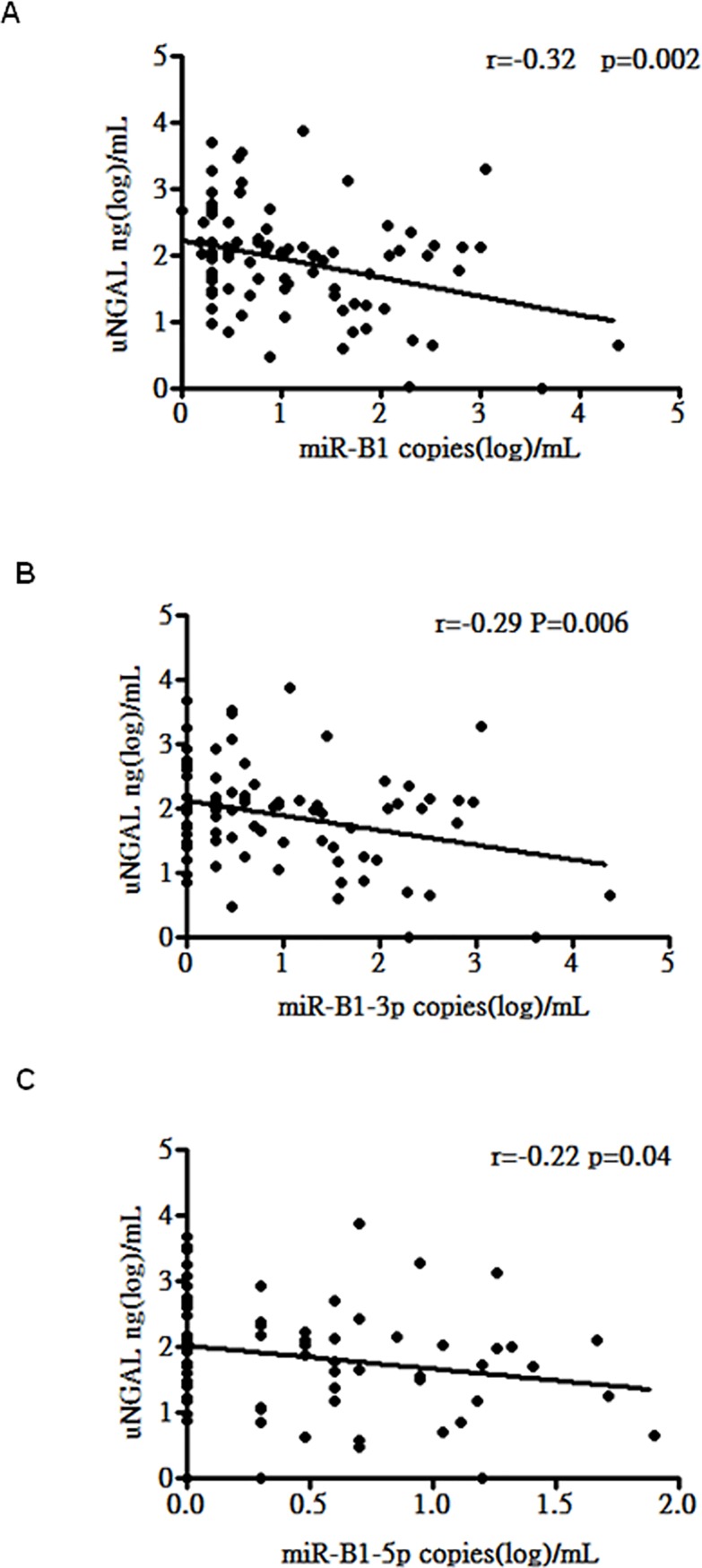
Correlations between uNGAL and urinary miR-B1 levels in patients with SLE. A. Correlation between uNGAL level and urinary level of total miR-B1. B. Correlation between uNGAL level and urinary level of miR-3p-B1. C. Correlation between uNGAL level and urinary level of miR-5p-B1.

As patients with LN had higher uNGAL levels and lower urinary BKV miR-B1 levels, we further assessed whether there was an association between the two parameters in patients with SLE. As shown in Fig 1, urinary levels of miR-B1 were negatively correlated with uNGAL expression (r = -0.33, p = 0.002) (Fig 1A). Further subgroup analysis also revealed that urinary levels of miR-B1-5p and 3p and uNGAL were negatively correlated (Fig 1B and 1C). These results suggest that urinary BKV miR-B1 levels negatively correlate with uNGAL levels in patients with SLE.

### The uNGAL level is a predictor of LN and correlates with baseline eGFR in patients with SLE

**Fig 2:**
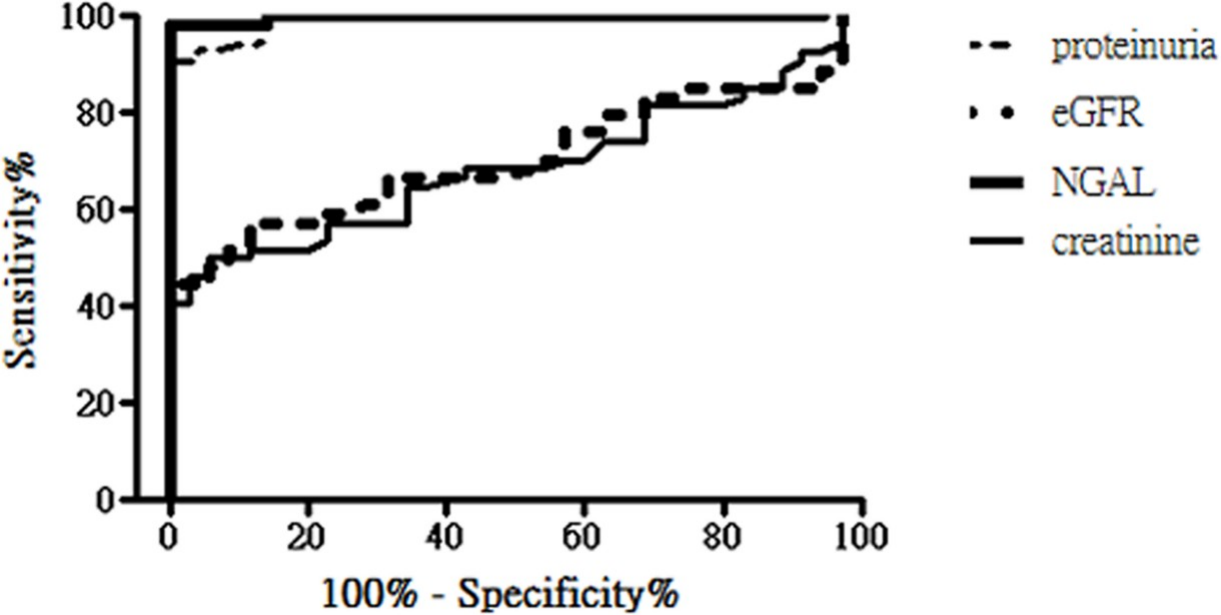
The area under the receiver operating characteristic (ROC) curves (AUC) constructed for uNGAL, proteinuria, eGFR and serum creatinine levels in predicting the presence of biopsy-proven lupus nephritis in patients with systemic lupus erythematosus. The AUC of uNGAL (solid line) was 0.99 (p < 0.0001). The AUC of proteinuria, eGFR and serum creatinine was 0.97 (p<0.0001), 0.71 (p = 0.0011) and 0.69 (p = 0.0028), respectively.

uNGAL has been reported as a useful biomarker for predicting LN severity.[23] In the current study, patients in the LN group had significantly higher uNGAL levels than patients in the non-LN group (Table 1). ROC curve analysis was used to determine the uNGAL levels to discriminate patients with LN from patients without LN. As shown in Fig 2, the AUC of uNGAL was 0.99 (p < 0.0001); at the cutoff value of 80 ng/mL, it had 98% sensitivity and 100% specificity, indicating high predictability for LN. The AUC of uNGAL was similar to that of proteinuria but higher than that of baseline eGFR and serum creatinine level (the AUC: 0.69, p < 0.005) in predicting the presence of LN. This suggests that baseline uNGAL level is a better biomarker in predicting the presence of biopsy-proven LN than baseline eGFR and serum creatinine level.

**Fig 3:**
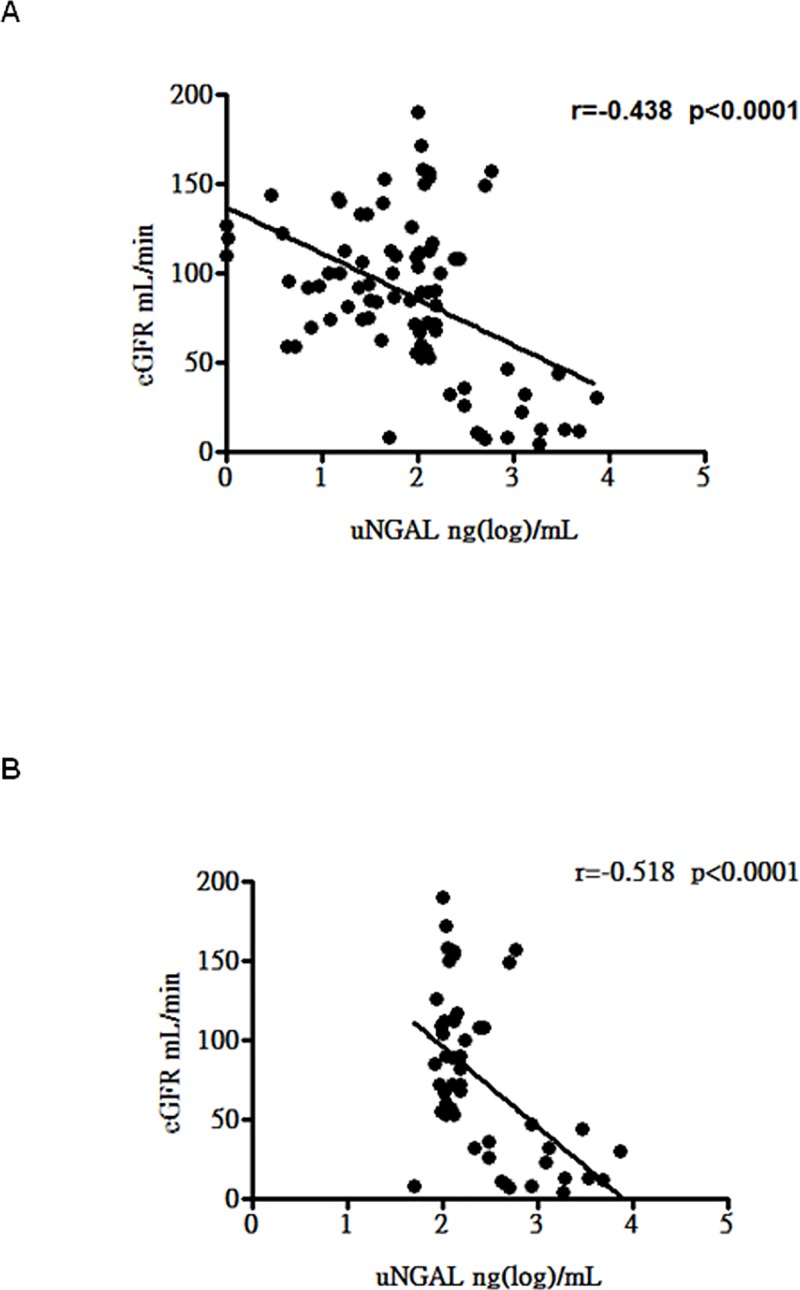
Correlations between eGRF and uNGAL level in all SLE patients and patients with LN. A. Correlation between eGRF and uNGAL level in all SLE patients. B. Correlation between eGFR and uNGAL level in patients with LN.

To further assess the association between uNGAL levels and LN severity, the correlation between baseline eGFR and uNGAL levels was determined. As shown in Fig 3A, the correlation between uNGAL levels and baseline eGFR was statistically significant in all patients with SLE (r = -0.408, p < 0.0001). Moreover, there was a significant correlation between uNGAL levels and baseline eGFR in patients with LN (r = -0.398, p = 0.0038) (Fig 3B).

### uNGAL is a useful predictor for the development of CKD in patients with SLE

During the 54-month follow-up, 14 patients with SLE (7%) had CKD, which was defined as a eGFR < 60 mL/min persisting for >3 months regardless of the presence or absence of histological kidney damage or proteinuria. There was a significant difference in the development of CKD in patients with versus those without LN (14 (26%) vs. 0 (0%), respectively; p = 0.030). Among these 14 patients, 6 progressed to ESRD (six (11%) vs. 0 (0%), respectively; p < 0.001).

Logistic regression analysis was used to identify the risk factors for the development of CKD. Univariate Cox regression analysis showed that the presence of LN, baseline creatinine and eGFR levels, use of prednisolone, and uNGAL level were risk factors for the development of CKD (Table 2). Multivariate Cox regression analysis identified that uNGAL was the only risk factor of CKD development. (Table 3)

**Table 2:** Hazard ratio of the occurrence of CKD on univariate Cox regression analysis.

	Hazard ratio	Univariate 95% confidence interval	P value
Presence of LN	0.33	0.12–0.90	0.029
Age	0.98	0.95–1.01	0.34
Hemoglobin (g/mL)	0.86	0.71–1.03	0.10
Creatinine (mg/dL)	1.44	1.20–1.71	<0.001
eGFR (mL/min)	0.98	0.97–0.99	<0.001
Albumin (g/dL)	0.96	0.60–1.54	0.86
Low C3 (%)	1.25	0.53–2.95	0.62
Low C4 (%)	1.72	0.74–4.00	0.21
Positive anti-dsDNA (%)	1.02	0.45–2.31	0.97
Proteinuria (mg/dL)	1.00	1.00–1.00	0.06
Potent immunosuppressants^a^ (%)	0.53	0.21–1.36	0.19
Prednisolone (mg/day)	1.05	1.01–1.08	0.006
Urinary NGAL (ng/mL)	1.00	1.00–1.00	<0.001
Urinary BKV copies (log)/mL	0.93	0.63–1.38	0.71
BKV miR-B1-5p copies (log)/mL	0.58	0.24–1.36	0.21
BKV miR-B1-3p copies (log)/mL	0.80	0.50–1.28	0.35

Anti-dsDNA, anti-double-stranded DNA antibody; BKV, polyomavirus BK; CKD, chronic kidney disease; LN, lupus nephritis; NGAL, neutrophil gelatinase-associated lipocalin

^a^Including mycophenate mofetil, azathioprine, and cyclophosphamide

**Table 3:** Hazard ratio of the occurrence of CKD on multivariate Cox regression analysis.

	Hazard ratio	Multivariate 95% confidence interval	P value
Presence of nephritis	0.90	0.24–3.27	0.87
eGFR (mL/min)	0.98	0.96–1.00	0.07
Creatinine (mg/dL)	0.91	0.59–1.40	0.67
Prednisolone (mg/day)	1.04	0.98–1.10	0.17
Urinary NGAL (ng/mL)	1.00	1.00–1.00	0.014

CKD, chronic kidney disease; eGFR, estimated glomerular filtration rate; NGAL, neutrophil gelatinase-associated lipocalin

## Discussion

The current study demonstrated that patients with LN had higher urinary BKV loads than those without LN. BKV reactivation is commonly discovered in immunocompromised kidney transplant patients. In non-transplant patients, BK viruria is frequently discovered in patients with SLE as they have impaired immunity and are further immunosuppressed by the use of potent immunosuppressants. The prevalence of BK viruria has been reported to range from 10% to 71%.[13, 24] LN, which affects 50% of patients with SLE, is a major factor for mortality in patients with SLE and frequently progresses to CKD and ESRD. Although several studies have analyzed the characteristics of patients with SLE with BK viruria, patients with BK viruria by LN status have yet to be compared. The current study showed a higher urinary BKV load in the LN group than in the non-LN group, suggesting a more seriously disturbed or suppressed immunity in patients with LN due to active renal flares, which was reflected by the lower serum C3 and C4 levels, higher number of patients with anti-dsDNA and a greater degree of proteinuria, increasing dose of steroids, and more potent immunosuppressant use (Table 1). It has also been reported that some inflammatory cytokines such as tumor necrosis factor (TNF) and interleukin 6 can facilitate replication of the JC virus, which bears 70% homology to BKV.[25, 26] We speculate that in comparison to the non-LN kidney, the inflammatory microenvironment in the LN kidney may promote BKV replication, thus causing a higher urinary BKV load. Another cause of a higher urinary BKV load in the LN group might be greater BKV shedding and release from damaged tubular cells into the urine. In the kidney transplant recipients, the presence of the BKV TAg-stained decoy cells that shed from damaged uroepithelial cells into the urine is an important marker of severe BKV infection.[27] Compared to patients without LN, those with LN might have more damaged tubular cells, as reflected by the higher uNGAL levels, thus leading to a higher urinary BKV load. As the use of immunosuppressants can accelerate BKV replication, urinary BKV load in SLE patients without the use of immunosuppressive therapy was measured. A significantly higher urinary BKV load was still observed in the LN group when compared to that in the non-LN group.

Rekvig et al. demonstrated that BKV reactivation in SLE was correlated with the production of autoantibodies recognizing dsDNA and SLE activity in animal models.[28] We previously showed an association between impaired renal function and a high urinary BKV load in kidney transplant recipients.[21] In this study, we did not find a correlation between urinary BKV load and eGFR in patients with SLE. There was also no correlation between urinary BKV load and uNGAL level. Furthermore, urinary BKV load did not predict the development of CKD. These results suggest that, unlike in kidney transplant recipients, a higher urinary BKV load is not the major cause of impaired renal function in LN patients but may only reflect an immunosuppressive status and tubular damage.

BKV-encoded microRNAs were reportedly detected in the urine of kidney transplant recipients and suggested to be a biomarker of PVAN.[18] In this study, we found that mean urinary BKV miR-B1-5p level was significantly lower in the LN group than in the non-LN group. In contrast, urinary BKV miR-B1-3p level was not significantly different between groups. The expression levels of viral microRNA 3p and 5p commonly differ because the more stable strand becomes dominant and the less stable strand is susceptible to degradation. We previously demonstrated that BKV miR-B1 suppressed viral replication in cultured cells, leading to negative autoregulation. In this study, urinary BKV miR-B1-3p and -5p were not significantly correlated with urinary BKV loads, suggesting that an increase in urinary BKV load in patients with LN was not directly attributed to a reduction in BKV miR-B1–mediated negative autoregulation.

Some virus-encoded miRNAs are known to target host genes, affect host immune regulation, and intervene in host cell growth.[29] As miRNAs can be implicated in disease pathogenesis and remain undegraded in the body and tissue fluids, they can be used as biomarkers for some diseases.[30] In patients infected with chronic EBV, the plasma levels of the EBV-encoded miRNA profiles had a stronger predictive value to discriminate between active and inactive disease compared with viral DNA levels.[31] The mean urinary BKV miR-B1-5p level was lower in the LN group and negatively correlated with the uNGAL level, suggesting the potential use of a reduced urinary BKV miR-B1-5p level as a marker of kidney damage in patients with SLE. Verifying whether urinary BKV miR-B1-5p level can be used as a biomarker to replace kidney biopsy to predict LN requires a larger-scale study. Our unpublished data showed that introduction of BKV miR-B1 into renal proximal tubular cells, HRPTECs, reduced tumor necrosis factor-alpha-induced production of NGAL (data not shown). This suggests that BKV miRNAs suppress NGAL production in cell culture system. Nevertheless, whether there is a causal relationship between a BKV miR-B1 reduction and uNGAL level requires further investigation.

This study demonstrated that mean uNGAL level was significantly higher in the LN group than in the non-LN group. Using 80 ng/mL as a cutoff value, uNGAL had high predictability for LN with 98% sensitivity and 100% specificity. Compared with eGFR and serum creatinine level, baseline uNGAL level is a better biomarker in predicting the presence of biopsy-proven LN. Moreover, the baseline uNGAL levels were negatively correlated with baseline eGFR. Several studies have shown that uNGAL is a reliable biomarker for both acute kidney injury and CKD in patients with SLE.[32] NGAL is possibly involved in the pathogenesis of LN as the kidney injury of antiDNA antibody-induced nephritis was reduced in NGAL-knockout mice compared to wild-type mice.[33] Because NGAL can promote inflammation and cause tubular apoptosis^8^ and uNGAL level correlates with the severity of renal damage,[34] higher uNGAL levels in patients with SLE with LN suggest more severe kidney damage than in those without LN.

Although baseline uNGAL level was correlated with eGFR, multiple logistical regression analysis showed that, during the 54-month follow-up period, baseline uNGAL level was the only independent risk factor for the development of CKD, while baseline anti-dsDNA, serum creatinine levels, and eGFR were not. The reasons why baseline uNGAL level was a better predictor for the development of CKD than baseline serum creatinine level and eGFR are unclear. We assume that uNGAL reflected more severe and prolonged tubular damage that had jeopardized compensation mechanisms and caused irreversible change than baseline eGFR and serum creatinine did. In addition, several studies have demonstrated that the increase in uNGAL precedes the increase in serum creatinine level.[35] The increase in uNGAL level might occur earlier than the rise of serum creatinine level, which would partially explain our result. The early prediction of the development of CKD is crucial for patients with SLE because the more potent immunosuppressants may require changing in these patients. Our study suggests that uNGAL is a useful predictor of the development of CKD, even better than proteinuria, serum creatinine level and eGFR. Torres-Salido et al. demonstrated that uNGAL was the only parameter correlated with the activity score of renal biopsies and a good predictive biomarker of renal flare in patients with LN.[23] Hinze et al. showed that a significant increase in uNGAL/creatinine levels was observed 3 months prior to LN deterioration.[36] In line with these studies, this study also suggests that uNGAL is a good predictive biomarker of CKD development.

In conclusion, the present study demonstrated that uNGAL level was increased in patients with LN and correlated with impaired renal function in patients with SLE. Increased uNGAL level was a good predictive biomarker of CKD development. An increased urinary BKV load and reduced urinary BKV miR-B1-3p level were found in patients with LN. As there was a negative correlation between urinary miR-B1-3p and uNGAL levels, a decreased urinary BKV miR-B1 level in combination with an increased uNGAL level could be a potential indicator of renal damage in patients with SLE.
